# Cell-Mediated and Peptide-Based Delivery Systems: Emerging Frontiers in Targeted Therapeutics

**DOI:** 10.3390/pharmaceutics17121597

**Published:** 2025-12-11

**Authors:** Eszter Erdei, Ruth Deme, Balázs Balogh, István M. Mándity

**Affiliations:** 1 Artificial Transporter Research Group, Institute of Materials and Environmental Chemistry, HUN-REN Research Centre for Natural Sciences, Magyar Tudósok krt. 2, H-1117 Budapest, Hungary; erdei.eszter@stud.semmelweis.hu; 2 Institute of Organic Chemistry, Semmelweis University, Hőgyes Endre u. 7, H-1092 Budapest, Hungary; deme.ruth@semmelweis.hu (R.D.); balogh.balazs@semmelweis.hu (B.B.)

**Keywords:** cell-mediated, peptide, drug delivery, nanotechnology, BBB, medicine

## Abstract

**Background/Objectives:** Cell-mediated and peptide-assisted delivery systems have emerged as powerful platforms at the intersection of chemistry, nanotechnology, and molecular medicine. By leveraging the intrinsic targeting, transport, and signaling capacities of living cells and bioinspired peptides, these systems facilitate the delivery of therapeutic agents across otherwise restrictive biological barriers such as the blood–brain barrier (BBB) and the tumor microenvironment. This review aims to summarize recent advances in engineered cell carriers, peptide vectors, and hybrid nanostructures designed for enhanced intracellular and tissue-specific delivery. **Methods:** We surveyed recent literature covering molecular design principles, mechanistic studies, and in vitro/in vivo evaluations of cell-mediated and peptide-enabled delivery platforms. Emphasis was placed on neuro-oncology, immunotherapy, and regenerative medicine, with particular focus on uptake pathways, endosomal escape mechanisms, and structure–function relationships. **Results:** Analysis of current strategies reveals significant progress in optimizing cell-based transport systems, peptide conjugates, and multifunctional nanostructures for the targeted delivery of drugs, nucleic acids, and immunomodulatory agents. Key innovations include improved BBB penetration, enhanced tumor homing, and more efficient cytosolic delivery enabled by advanced peptide designs and engineered cellular carriers. Several platforms have progressed toward clinical translation, underscoring their therapeutic potential. **Conclusions:** Cell-mediated and peptide-assisted delivery technologies represent a rapidly evolving frontier with broad relevance to next-generation therapeutics. Despite notable advances, challenges remain in scalability, manufacturing, safety, and regulatory approval. Continued integration of chemical design, molecular engineering, and translational research will be essential to fully realize the clinical impact of these delivery systems.

## 1. Introduction

Targeted intracellular delivery remains an important challenge in modern pharmacology and gene therapy. Classical nanocarriers, though versatile, often fail to replicate the precision, biocompatibility, and dynamic responsiveness of biological systems. The emergence of cell-mediated and peptide-based vectors has introduced a bioinspired paradigm, integrating molecular recognition, receptor-mediated transcytosis, and immune cell homing mechanisms into drug delivery design.

Cell-mediated systems—using leukocytes, macrophages, dendritic cells (DCs), or stem cells as biological shuttles—can actively navigate physiological barriers and deliver therapeutic cargos to otherwise inaccessible tissues. Similarly, cell-penetrating peptides (CPPs), receptor-targeting sequences, and self-assembling peptidomimetics have been developed to mimic or harness cellular communication pathways [1,2,3]. Cell-mediated delivery systems and CPPs represent two complementary strategies designed to overcome biological barriers and achieve efficient intracellular transport of therapeutic agents [4,5,6]. CPPs are short peptide sequences capable of translocating across cellular and other membranes, thereby facilitating the direct delivery of diverse cargos such as small molecules, nucleic acids, proteins, or nanoparticles into target cells and compartments within [7,8,9]. In contrast, cell-mediated delivery systems utilize living cells—such as immune cells, stem cells, or macrophages—as biological carriers that can naturally home to disease sites and transport therapeutic payloads in a physiological context [10,11,12,13]. The combination of these two approaches offers a synergistic advantage: CPPs can enhance the loading efficiency of therapeutic cargos into carrier cells and promote controlled intracellular release, while the inherent homing ability of carrier cells improves biodistribution and accumulation in the targeted tissues. Integrating CPPs into cell-mediated systems thus holds great promise for achieving precise, efficient, and safe therapeutic delivery in complex biological environments [14,15,16,17].

Immune-modulating peptides (IMPs) and cell-mediated delivery systems form a promising fusion in the development of next-generation immunotherapies [18,19]. IMPs are short peptide sequences that regulate immune responses by interacting with receptors or signaling pathways involved in inflammation, immune activation, or tolerance [20]. They can either stimulate immune functions—for example, enhancing antigen presentation or T-cell activation—or suppress pathological inflammation in autoimmune and inflammatory diseases [21]. Despite their high potency and selectivity, IMPs often hindered by limitations such as enzymatic degradation, short circulation half-life, and insufficient tissue specificity. Cell-mediated delivery systems, including immune cells, stem cells, and engineered exosomes, provide a solution contributing as living carriers that naturally migrate to immune-relevant sites [22,23]. Incorporating IMPs into such systems enhances targeted delivery, protects the peptides from degradation, and enables controlled or stimulus-responsive release in the microenvironment of immune activation [24,25,26]. Conversely, certain IMPs can modulate the phenotype or function of the carrier cells themselves, further optimizing therapeutic outcomes [27]. The integration of immune-modulating peptides with cell-mediated delivery platforms thus represents a synergistic approach that unites precise immunoregulation with biologically guided targeting and sustained therapeutic action [28,29].

RGD peptides, containing the arginine–glycine–aspartic acid (RGD) motif, are among the most studied targeting ligands due to their high affinity for integrin receptors, particularly αvβ3 and αvβ5, which are overexpressed on angiogenic endothelial cells (ECs) and various tumor types [30,31,32]. These peptides facilitate selective recognition and adhesion to integrin-rich tissues, enabling enhanced targeting in cancer therapy, tissue regeneration, and vascular repair [33,34,35,36]. Cell-mediated delivery systems—such as macrophages, mesenchymal stem cells, and immune cells—possess innate homing abilities that can be further optimized through RGD functionalization [37]. Conjugating or decorating carrier cells, nanoparticles, or their surface vesicles with RGD peptides can improve cell adhesion, transmigration across endothelial barriers, and accumulation at pathological sites characterized by integrin overexpression [38,39]. Conversely, RGD–integrin interactions can modulate the activation state and migration behavior of the carrier cells themselves, refining their therapeutic performance [40]. The integration of RGD peptides into cell-mediated delivery systems thus provides a dual advantage: enhanced biological targeting precision through receptor-mediated recognition and improved cellular navigation within complex tissue microenvironments [41,42]. This interplay holds significant potential for advancing targeted cancer therapies, regenerative medicine, and vascular tissue engineering [43,44,45].

Together, these systems redefine the concept of “smart” delivery: not merely passive transporters but active biological participants in therapeutic intervention.

## 2. Peptide-Based Delivery Systems

### 2.1. Cell-Penetrating Peptides and Gene Transfer

CPPs such as trans-activator of transcription (TAT), penetratin, or Ras-related protein (RALA) have long been recognized as potent translocation motifs. Recent advances have refined their selectivity and efficiency through modular design. Ding et al. developed neurokinin-1 receptor (NK1R) targeting vectors (SP-PEG_4_-K(C_18_)-(LLHH)_3_-R9) combining cell-penetrating, targeting, and endosomal escape domains to achieve specific transfection of glioma cells and successful blood-brain barrier (BBB) crossing in zebrafish models. The work exemplifies receptor-mediated uptake coupled with peptide self-assembly into gene-delivery nanocomplexes, achieving >30% higher transfection than the transfection reagent Lipofectamine in NK1R-positive cells [46].

Similarly, Jeyarajan et al. engineered a heregulin-α–NLS fusion protein targeting HER2/3-overexpressing breast cancer cells, enabling nucleus-specific plasmid DNA delivery [47]. By modularly combining a receptor ligand with a nuclear localization signal, the system attained selective uptake in MDA-MB-453 cells while avoiding non-target tissues.

In parallel, Neves et al. optimized the cationic RALA peptide/pDNA system for p53 delivery, demonstrating that the N/P ratio can fine-tune condensation, charge, and gene-expression efficiency, directly modulating apoptosis in cancer cells [48]. These studies collectively illustrate that fine molecular engineering—through charge balance, lipidation, and receptor ligands—translates into controllable intracellular trafficking and therapeutic output.

### 2.2. CPPs in Vaccine Development

**Figure 1:**
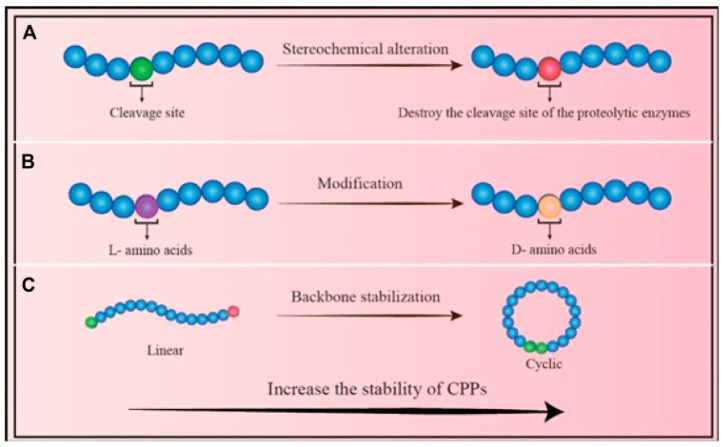
Approaches to Enhance CPP Stability: (**A**) Stereochemical modification: Altering the chemical configuration of amino acid residues reduces the enzymatic susceptibility of CPPs. (**B**) Amino acid substitution: Replacing natural residues with alternative isomers can significantly improve CPP stability. (**C**) Backbone cyclization: Converting linear CPPs into cyclic forms enhances resistance to enzymatic degradation and increases overall structural stability [49].

CPPs have also redefined vaccine formulation by enabling cytosolic delivery of protein and peptide antigens. Hasannejad-Asl et al. comprehensively reviewed CPP-conjugated vaccines, highlighting their capacity to enhance cross-presentation, antigen processing, and T-cell activation [49]. Strategies to enhance CPP stability are presented in Figure 1.

Notably, Z12-conjugated (small nucleolar RNA) protein vaccines promoted multi-epitopic CD4^+^/CD8^+^ responses against brain tumors, while LAH4-adjuvanted protein vaccines induced strong CTL immunity via endosomal acidification and proteasomal processing [50]. Pep-1/E7 nanoparticles likewise elicited Th1-biased protective responses in HPV16 tumor models comparable to Freund’s adjuvant [51].

Collectively, these CPP-based vaccines merge efficient intracellular transport with immunomodulation, yielding self-adjuvating platforms that circumvent the limitations of traditional emulsions or liposomes.

### 2.3. Peptidomimetic and Self-Assembled Nanostructures

Beyond natural sequences, peptidomimetics offer enhanced stability and tunable self-assembly. In a recent review, Jabbari et al. summarized the peptidomimetic self-assembled nanoparticles (NPs) that integrate biological recognition motifs with structural robustness [52]. These NPs serve as scaffolds for sustained antigen presentation, tumor targeting, and gene delivery while resisting enzymatic degradation. Their self-assembly—driven by hydrophobic, hydrogen-bonding, and electrostatic interactions—provides an elegant route to multivalent nanostructures with programmable morphology and function.

Table 1 summarizes seven peptide-based delivery systems, all of which show generally strong in vitro efficacy and low toxicity across various cell types and models. Their scalability is usually feasible through solid-phase peptide synthesis, though most systems have not yet been evaluated at true industrial or GMP scale. Overall, the technologies remain at early preclinical stages, with most evidence limited to in-vitro studies and small animal experiments, and no peptide-based systems yet in clinical trials.

## 3. Cell-Mediated Delivery Systems

### 3.1. Engineered Immune Cells as Therapeutic Couriers

Leveraging the intrinsic homing ability of immune cells has emerged as a powerful strategy for precision oncology. Wang et al. introduced monocyte-based photoactive nanoparticle delivery, where monocytes loaded with near-infrared (NIR) responsive polyacrylates (PAAs) achieved deep tumor penetration and dual photodynamic/photothermal ablation [53]. Metabolic surface glycoengineering with cyclic RGD peptides enhanced tumor migration and retention, enabling precise image-guided therapy.

Similarly, Liu et al. developed β-cyclodextrin/peptide-linked liposomes that release Matrix metalloproteinase-2 (MMP-2) inhibitors and photosensitizers sequentially within the tumor microenvironment [54]. This approach remodeled the tumor immune niche, increased NK-cell infiltration >100-fold, and yielded potent synergistic photo-immunotherapy—an exemplary demonstration of cell-microenvironment interplay for therapeutic benefit.

Mast cells, macrophages, and stem cells have also been investigated as carriers or trigger cells [55]. Their migratory behavior, cytokine release, and biocompatibility render them attractive vehicles for localized delivery of oncolytic, gene, or regenerative payloads.

### 3.2. Endothelial Cell-Mediated Vascular Repair

Zhou et al. introduced an elegant endothelial-cell-mediated gene delivery graft in which adhesive peptides with Arg–Glu–Asp–Val (REDV) motif capture ECs and trigger localized gene release through matrix metalloproteinase-cleavable linkers [56]. This responsive interface initiated selective adhesion, enzymatic activation, and in situ endothelialization of vascular grafts in vivo—illustrating how cellular microenvironment cues can be harnessed for regenerative biointerfaces.

Table 2 compares several endosomal-escape mechanisms—including proton-sponge buffering, direct membrane disruption, membrane fusion, and endosomal enzyme–responsive systems—highlighting their strengths, limitations, and current development status all of data with crucial importance for cell based delivery systems. Overall, while many strategies demonstrate strong mechanistic rationale and good in-vitro performance, most lack robust in-vivo validation and remain at early preclinical stages, with safety, specificity, and scalability posing the main challenges.

## 4. Overcoming the Blood–Brain Barrier

### 4.1. Nanomaterial and Peptide Strategies

**Figure 2:**
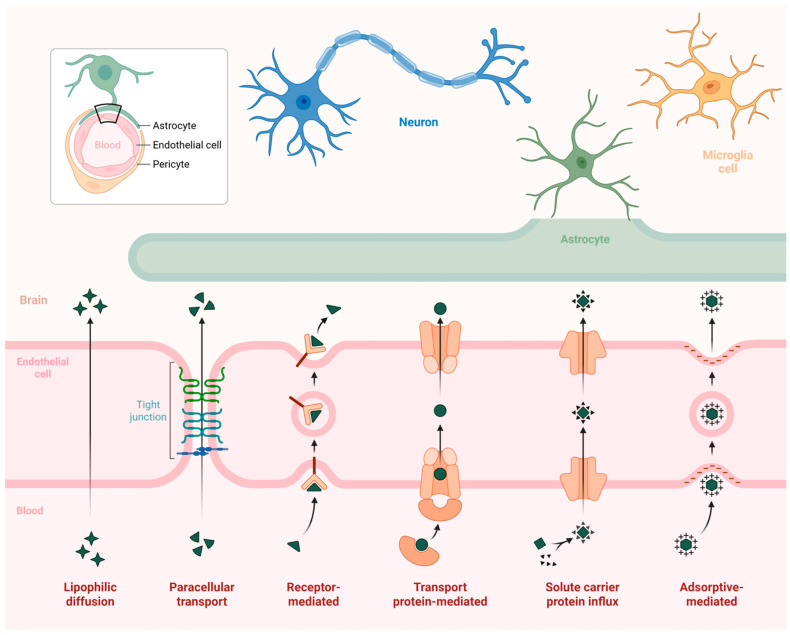
Mechanisms of Blood–Brain Barrier Permeation.

The BBB remains the principal obstacle in CNS therapeutics. As emphasized in a review by Patel and Xie, delivering strategies now extend beyond passive diffusion to receptor-mediated, adsorptive, and cell-mediated transcytosis [57,58]. Pathways of transport across the blood–brain barrier are shown in Figure 2. Nanocarriers equipped with CPPs or receptor-binding motifs (e.g., angiopep-2, transferrin) demonstrate promising BBB permeability, while cell-based transporters such as macrophages or stem cells exploit natural trafficking routes to deliver nanodrugs to diseased brain regions [59].

Mathupala highlighted the potential of CPP-siRNA duplexes for non-invasive CNS delivery, proposing molecular constructs capable of crossing the BBB systemically [60]. Similarly, Man et al.’s PEG_12_-KL4 peptide enabled pulmonary delivery of EGFR/PD-L1-targeted siRNAs, with efficient knockdown and enhanced T-cell–mediated cytotoxicity—offering a blueprint for nucleic acid delivery through accessible epithelial interfaces [61].

### 4.2. Bioactive Nanoparticles for Neurodegenerative Diseases

**Figure 3:**
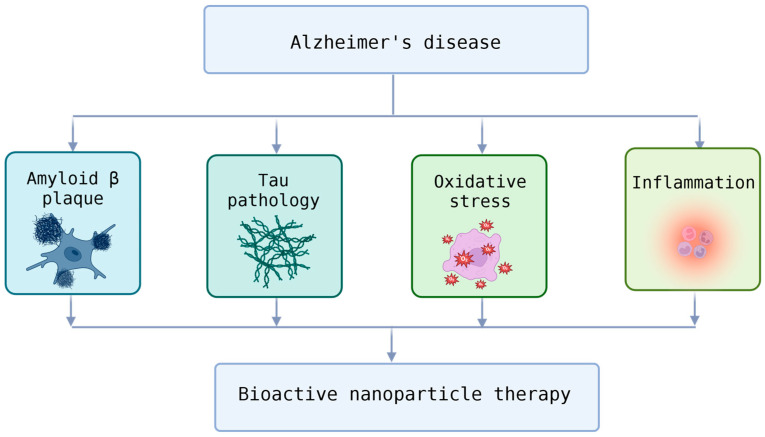
Therapeutic Targets in Alzheimer’s Disease and the Potential of Bioactive Nanoparticle Therapy.

Kshirsagar et al. reviewed bioactive compound-loaded nanoparticles addressing Alzheimer’s pathology (shown in Figure 3) through antioxidant and anti-inflammatory mechanisms [62]. Importantly, cell-mediated and transcytosis-based BBB transport emerged as critical determinants of efficacy, reinforcing the concept that combining nanotechnology with biological transport systems yields the most promising neurotherapeutic outcomes.

## 5. Immunotherapeutic Integration

Wang and Wang outlined the molecular rationale for intracellular peptide delivery into DCs to stimulate pattern-recognition receptor signaling and augment cytotoxic T-cell responses [63]. Kimura et al. expanded this principle using endosomal sorting complexes required for transport (ESCRT) mediated extracellular vesicles for efficient antigen loading and CTL activation—highlighting a convergence between cell-derived vesicles and engineered peptide vectors in vaccine design [64].

The synergy between photo-immunotherapy as in microenvironment-/light-responsive bio-nanosystems (MLRNs) and immune-cell engagement (e.g., NK or CD8^+^ recruitment) underscores a transformative shift from single-agent cytotoxicity toward immune-coordinated multimodal therapy.

Table 3 outlines multiple cell-uptake mechanisms—including clathrin-mediated endocytosis, caveolae-mediated uptake, macropinocytosis, phagocytosis, and direct translocation—highlighting their pathways, cargo preferences, and biological constraints. While each mechanism offers unique advantages for targeting specific cell types or achieving efficient internalization, they also come with limitations such as size restrictions, dependence on energy or receptor pathways, and variable reliability across different biological contexts. Overall, selecting the optimal uptake route requires balancing specificity, efficiency, and compatibility with the delivered cargo.

## 6. Ocular and Localized Delivery

Beyond systemic delivery, cell-mediated concepts extend to ocular surfaces, where the application of traditional eye drops is limited by poor retention. In a study by He et al. a soluble microneedle patch loaded with immunogenic PKHB1 peptide penetrated corneal barriers, released the peptide sustainably, and elicited CD8^+^ T-cell–mediated antiviral responses against HSV-1 keratitis [65]. The system exemplifies how microengineered matrices can recreate cell-mediated immunity in confined tissues.

## 7. Design Principles and Structure–Function Insights

### 7.1. Molecular Modularity

Across all platforms, the efficiency and precision of cell-mediated and peptide-based delivery systems depend on their modular molecular architecture, in which individual functional domains perform distinct yet complementary tasks to achieve targeted, controlled, and biocompatible therapeutic delivery [66].

Targeting domains (e.g., Substance P, heregulin, RGD, REDV) ensure receptor-specific recognition and guide the delivery system toward the intended tissue or cell type. Such motifs exploit overexpressed receptors or adhesion molecules—Substance P targets NK1Rs in glioma cells, heregulin interacts with HER2/3 in breast cancer, while RGD and REDV sequences mediate integrin or endothelial cell adhesion, respectively [67].

**Figure 4:**
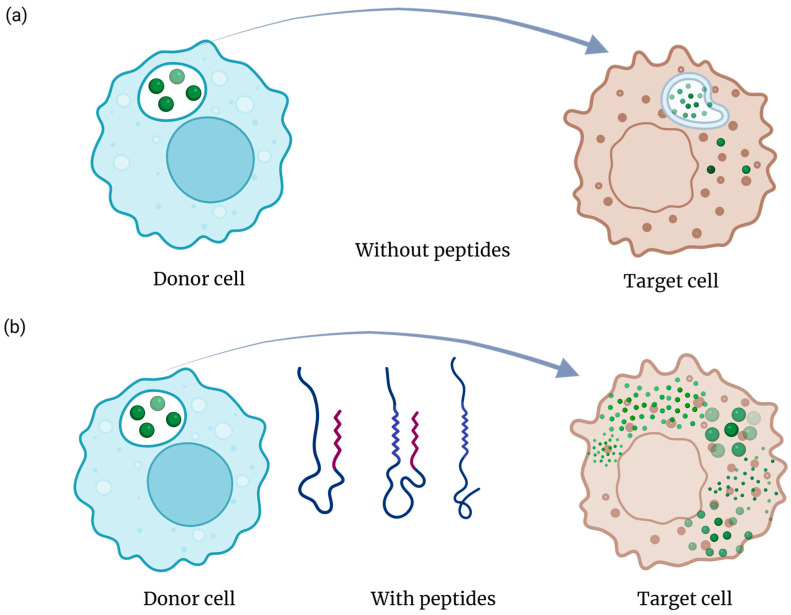
Sematic representation of endosomal escape in the absence (**a**) and presence (**b**) of peptide sequences. Peptides can enhance the efficacy of CMDS by enhancing the endosomal escape of cargo molecules.

Cell-penetrating peptide (CPP) or fusogenic domains facilitate membrane translocation and endosomal escape, enabling intracellular delivery of nucleic acids, proteins, or small molecules (Figure 4). Classical CPPs like TAT, penetratin, or RALA promote uptake through electrostatic interactions, while pH-sensitive fusogenic sequences such as LAH4 or HA2 undergo conformational transitions that destabilize endosomal membranes and release the cargo into the cytosol. GraphCPP leverages graph neural networks to learn residue–residue interaction patterns within peptide sequences, enabling far more nuanced CPP prediction than traditional feature-based classifiers. In benchmark evaluations, GraphCPP improves prediction accuracy by roughly 8–15% over conventional SVM and random-forest models and shows markedly higher precision for borderline or atypical CPPs. Its graph-based embedding also enhances generalization, reducing false positives by capturing structural motifs missed by linear sequence descriptors [68,69].

Responsive linkers—including pH-, MMP-, or redox-cleavable motifs—provide spatiotemporal control over activation and release. For instance, MMP-sensitive linkers allow for selective drug liberation within protease-rich tumor microenvironments, while disulfide-based redox linkers respond to intracellular glutathione gradients for cytosolic release of nucleic acids or peptides [70].

Hydrophobic anchors or PEG linkers enhance physicochemical stability and pharmacokinetic tunability. Hydrophobic tails such as stearic acid or cholesterol promote self-assembly into nanoparticles and strengthen membrane affinity, whereas PEGylation improves solubility, reduces immune recognition, and prolongs systemic circulation [71].

This modular design paradigm allows rational adaptation of vectors to distinct therapeutic contexts—in nucleic acid delivery, protein vaccination, or immune stimulation—by enabling the systematic optimization of each structural component. Through deliberate combination of targeting, translocation, responsiveness, and stabilization modules, these hybrid systems evolve from simple carriers into multifunctional, adaptive platforms capable of precise spatiotemporal control and enhanced therapeutic efficacy.

### 7.2. Cell Interactions and Microenvironment Sensitivity

Effective cell-mediated and peptide-based delivery systems must not only carry their therapeutic payloads but also interface intelligently with the biological microenvironment. The performance of these systems depends on their ability to recognize, respond to, and exploit the physicochemical and biochemical cues of target tissues. This sensitivity governs biodistribution, internalization, and controlled release, linking molecular design to cellular behavior and tissue specificity.

Microenvironment-responsive activation allows selective function at the disease site. Many tumors and inflamed tissues are characterized by acidic pH, elevated enzyme activity (e.g., matrix metalloproteinases, cathepsins), oxidative stress, or abnormal cytokine profiles. Delivery vectors incorporating pH-sensitive residues or MMP-cleavable peptide linkers exploit these pathological conditions to trigger cargo release or surface activation specifically within the diseased milieu. For example, MMP-2-responsive nanoconjugates enable site-specific release of photosensitizers and immunomodulators in tumor tissue, enhancing therapeutic precision while minimizing systemic toxicity [72].

Receptor-mediated uptake and cell selectivity arise from the interplay between targeting ligands and overexpressed membrane receptors. Peptide ligands such as RGD, REDV, Substance P, or heregulin interact with integrins, endothelial adhesion receptors, or growth factor receptors, enabling selective internalization by cancer, endothelial, or immune cells. The specificity of these interactions underpins the success of strategies such as NK1R-mediated glioma targeting and HER2/3-driven gene delivery in breast cancer [73].

**Figure 5:**
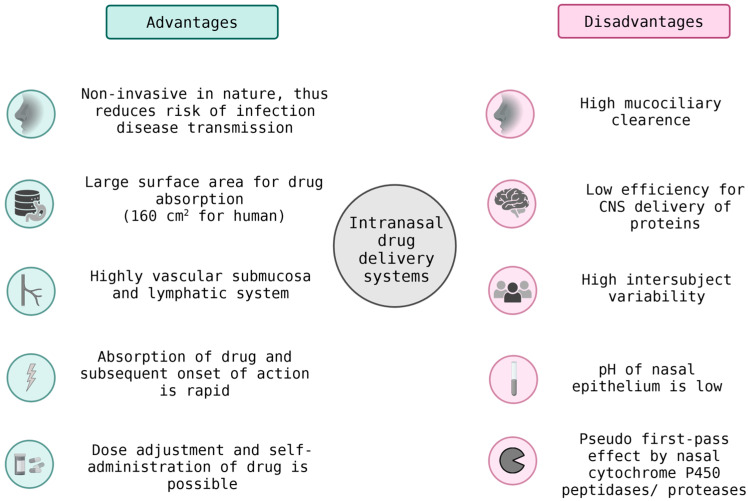
Intranasal Drug Delivery Systems: Benefits and Limitations in BBB Transport.

Cellular cooperation and transport mechanisms expand the reach of therapeutic systems beyond passive diffusion. Engineered immune or endothelial cells can actively ferry nanoparticles and peptide–drug conjugates across biological barriers such as the blood–brain barrier (BBB) or tumor stroma (Figure 5). Intranasal drug delivery provides a noninvasive route to bypass the BBB, enabling rapid and targeted delivery to the brain. While it offers reduced systemic side effects, its efficiency can be limited by mucociliary clearance and enzymatic degradation. Monocyte- or macrophage-based carriers, for instance, migrate to inflammatory or hypoxic regions, while endothelial cells can serve as living transfection platforms that promote localized angiogenesis or regeneration [74].

Dynamic biophysical interactions—including electrostatic attraction, hydrophobic partitioning, and membrane curvature effects—govern the initial contact between delivery systems and cell membranes. These interactions dictate internalization routes such as clathrin-mediated endocytosis, macropinocytosis, or lipid-raft–assisted uptake. Understanding and tuning these parameters allows rational modulation of uptake efficiency and intracellular trafficking pathways [75].

Immune compatibility and biological feedback are critical determinants of long-term efficacy. Peptide vectors and hybrid nanoparticles are necessary to avoid unwanted immune activation while engaging beneficial immune pathways when desired—for example, in vaccine delivery or cancer immunotherapy. Materials engineered with zwitterionic or PEGylated surfaces minimize the risk of nonspecific opsonization, whereas immunogenic peptides or adjuvant motifs can be incorporated deliberately to enhance antigen presentation and T-cell activation [76].

Overall, microenvironmental responsiveness transforms delivery systems from inert carriers into biointeractive entities capable of sensing and adapting to their surroundings. By integrating physicochemical responsiveness, receptor specificity, and cellular cooperation, such systems achieve precise control over localization, uptake, and release kinetics. This responsiveness is a defining feature of next-generation therapeutic platforms—where chemistry, materials science, and cell biology converge to achieve targeted and context-dependent drug delivery [77].

## 8. Translational and Regulatory Considerations

The transition of cell-mediated and peptide-based delivery systems from laboratory discovery to clinical application remains a formidable challenge. Despite impressive preclinical advances demonstrating potent therapeutic efficacy in vitro and in vivo, translation is often hindered by manufacturing complexity, product heterogeneity, and regulatory uncertainty. These systems, by their very nature—combining synthetic peptides, nanomaterials, and living cells—occupy a grey zone at the intersection of biologics, advanced therapy medicinal products (ATMPs), and nanomedicines, demanding specialized oversight frameworks and rigorous quality control [78,79].

Manufacturing and scalability are the main obstacles. The production of multifunctional peptide–nanoparticle conjugates or hybrid cell-based systems requires precise control over sequence fidelity, assembly conditions, and surface functionalization. Conventional batch synthesis can introduce variability in particle size, peptide density, or biological activity, complicating reproducibility and regulatory approval. Recent progress in continuous-flow solid-phase peptide synthesis (CF-SPPS) and automated purification offers a solution scalable and reproducible manufacturing under Good Manufacturing Practice (GMP) standards. Flow-based synthesis not only minimizes solvent use and reaction time but also allows for precise in-line monitoring of product quality—aligning with the green chemistry and quality-by-design (QbD) principles are favored by regulatory agencies. Using these technique, the process mass intensity value of the SPPS decreases to value of ca. 300, of which is in the order of magnitude of regular small-organic drug molecules. Importantly, by scale-up these values remains basically the same [80,81].

Characterization and standardization are critical for ensuring clinical reliability. Comprehensive physicochemical and biological profiling—including size distribution, charge, purity, stability, and in vitro bioactivity—must be supported by validated analytical assays. For hybrid systems, additional characterization of biodistribution, persistence, and immunogenicity is required to predict long-term safety. The integration of high-resolution imaging, quantitative mass spectrometry, and in vivo pharmacokinetic modeling aids in establishing robust comparability between production batches [82].

Immunological and biosafety evaluation remains a cornerstone of translational assessment. While peptide-based carriers generally exhibit low inherent immunogenicity, in combination with adjuvants, targeting ligands, or cellular components can lead to unpredictable immune responses. Regulatory agencies therefore demand extensive toxicological testing, cytokine-release profiling, and long-term immunosurveillance before first-in-human trials. In the context of cell-mediated delivery, additional attention must be given to cell persistence, tumorigenicity risk, and vector integration stability, particularly for engineered immune or stem cells [83].

Digital and AI-assisted optimization is emerging as a transformative tool for translation. Machine-learning algorithms can predict optimal peptide sequences, linker chemistries, and assembly parameters, reduce experimental cycles and help identifying lead candidates with more favorable manufacturing profiles. Combined with automated flow synthesis, such computational tools enable rapid, data-driven optimization for process control and traceability in accordance with regulatory expectations. GraphCPP leverages graph neural networks to learn residue–residue interaction patterns within peptide sequences, enabling far more nuanced CPP prediction than traditional feature-based classifiers. In benchmark evaluations, GraphCPP improves prediction accuracy by roughly 8–15% over conventional SVM and random-forest models and shows markedly higher precision for borderline or atypical CPPs. Its graph-based embedding also enhances generalization, reducing false positives by capturing structural motifs missed by linear sequence descriptors [69,84].

Clinical Translation and Regulatory Landscape of Cell–Peptide Hybrids have been examined in a limited but growing number of clinical trials, which have already demonstrated the translational potential of cell- and peptide-based delivery systems, particularly in oncology and immunotherapy. Early first-in-human studies with the tumor-targeting cell-penetrating peptide p28 (NSC745104) in adults and children with advanced solid or CNS tumors established acceptable safety and preliminary antitumor activity, illustrating that CPP-based agents can meet contemporary clinical and regulatory safety thresholds [85,86]. Dendritic cell-based vaccines and exosome formulations—often loaded with tumor peptides or antigens—have progressed to phase II and III trials, including IFN-γ–matured dendritic cell-derived exosomes (IFN-γ–Dex) in non-small cell lung cancer and DCVax-L in glioblastoma, highlighting the feasibility of complex cell-based products that integrate peptide antigens or peptide-decorated vesicles in a GMP-compliant manner [87,88,89]. In parallel, tumor-penetrating peptides such as certepetide (LSTA1) have entered later-stage trials in combination with chemotherapy, where they act as peptide-based enhancers of intratumoral drug penetration [90]. Selected examples are summarized in Table 4.

Table 4 describes key clinical-stage peptide- and dendritic-cell-based therapeutic modalities currently evaluated across oncology indications. It highlights five representative products—from tumor-penetrating and tumor-targeting cell-penetrating peptides (CPPs) to autologous dendritic-cell vaccines and exosome-based immunotherapies—covering phases I to III. Each entry outlines the cancer indication, ClinicalTrials.gov identifier, development phase, and a short note describing the mechanism or distinguishing features (e.g., p53-stabilizing CPP p28, antigen-loaded DC exosomes, tumor-lysate–pulsed DC vaccines, MSI-targeted immunotherapy, and the tumor-penetrating peptide LSTA1 that improves chemotherapeutic delivery). The table below provides an overview of advanced peptide-enabled and peptide-augmented clinical strategies in cancer therapy.

From a regulatory standpoint, cell–peptide hybrids sit at the interface between biologics, advanced therapy medicinal products (ATMPs), and nanomedicines, complicating classification and CMC (chemistry, manufacturing and controls). In the EU, many peptide–cell constructs will fall under the ATMP framework (Regulation (EC) No 1394/2007) as somatic cell therapy or combined ATMPs when cells are substantially manipulated or used for a non-homologous function [41,91,92]. This implies stringent centralized authorization, risk-based evaluation, and ATMP-specific GMP requirements, including full traceability, donor eligibility documentation, and long-term post-authorization follow-up. In the US, autologous carriers and ex vivo–modified cells are regulated as human cells, tissues, and cellular and tissue-based products (HCT/Ps) with additional cGTP/cGMP obligations under 21 CFR Part 1271 [93,94,95].

For cell–peptide hybrids in particular, several specific hurdles arise: (i) GMP for autologous products, where patient-specific manufacturing, donor screening, and chain-of-identity/chain-of-custody must be tightly controlled; (ii) potency assays that remain valid after peptide modification or repeated peptide loading—e.g., assays for antigen presentation, cytokine release, or target killing must be qualified and shown to correlate with clinical effect; and (iii) product heterogeneity and batch variability, since peptide decoration densities, cell activation status, or exosome cargo composition can change with starting material or process drift. Regulators increasingly expect a risk-based, ATMP-style control strategy with well-defined critical quality attributes (CQAs), in-process controls, and stability data tailored to these hybrid constructs [91,92,95]. Addressing these aspects early in development is essential to move cell-mediated and peptide-based delivery systems from promising preclinical tools to licensable therapies.

Regulatory convergence and framework adaptation are equally essential. The hybrid nature of these therapies calls for harmonization between biologics, device, and nanomedicine guidelines. Collaborative efforts between academia, industry, and medicines regulatory (EMA, FDA) and other authorities (e.g., ICH) are beginning to define specific standards for advanced combination products, including documentation of critical quality attributes (CQAs), batch release criteria, and post-market surveillance requirements [96].

In summary, while manufacturing reproducibility, immunological safety, and regulatory classification remain primary translational obstacles, emerging technologies such as continuous-flow peptide synthesis, automated analytics, and AI-driven optimization may offer viable solutions. Together with GMP-compliant cell engineering platforms, these innovations are paving the way toward scalable, standardized, and clinically translatable cell-mediated and peptide-based therapeutics, bridging the gap between experimental achievements and regulatory approval.

## 9. Outlook and Future Perspectives

The convergence of cell biology, peptide chemistry, and nanotechnology is resulting in the emergence of a new generation of adaptive therapeutic systems capable of dynamic interaction with their biological environment. These next-generation delivery platforms transcend the traditional concept of passive carriers, evolving instead into integrated, feedback-responsive therapeutic networks. Their development is expected to transform precision medicine, immunotherapy, and regenerative strategies through several interrelated innovations:

Hybrid bioartificial carriers integrating living cells with synthetic scaffolds will enable unparalleled precision in drug delivery and biological feedback control. By combining the navigational and homing properties of immune or stem cells with the stability and modularity of engineered nanomaterials, such systems can respond dynamically to local biochemical signals. Engineered macrophages, endothelial cells, or exosomes functionalized with peptide–polymer conjugates exemplify this hybrid approach, allowing sustained, on-demand release of therapeutic payloads and real-time adaptation to pathological changes [97].

Organelle-targeting peptides and multi-responsive linkers will extend the precision of delivery to the subcellular level. By incorporating sequences that direct cargos to mitochondria, lysosomes, or nuclei, researchers can achieve spatiotemporally controlled pharmacological effects. When combined with multi-stimuli–responsive linkers—triggered by pH, redox processes, enzymes, or light—these designs will enable hierarchical activation within specific organelles, improving therapeutic selectivity by minimizing off-target effects [98].

AI-driven sequence design and predictive modeling are poised to revolutionize the discovery and optimization of functional peptides. Machine learning (ML) algorithms and molecular dynamics (MD) simulations can correlate sequence features with membrane translocation, receptor binding, and toxicity profiles, thereby generating optimized CPPs or targeting ligands with enhanced selectivity and reduced immunogenicity. Integrating computational prediction with continuous-flow synthesis and high-throughput screening (HTS) will accelerate the translation of in silico designs into clinically viable candidates [99].

Multicellular therapeutic ecosystems, in which immune cells, endothelial cells, and synthetic vectors act cooperatively, may redefine drug delivery as a systems-level biological process. Instead of isolated carrier entities, future therapeutics could function as coordinated networks where different cellular and synthetic components perform complementary roles—such as targeting, immune modulation, and tissue repair—within a controlled, self-regulating framework. This cooperative paradigm mirrors natural tissue organization, promising improved therapeutic durability and safety [100].

Ultimately, cell-mediated and peptide-based delivery systems exemplify the evolution of drug delivery science from passive encapsulation toward interactive and intelligent therapeutics. By uniting the programmability of synthetic chemistry with the adaptability of living systems, these platforms hold the potential to deliver personalized, context-sensitive interventions for complex diseases—including cancer, neurodegeneration, and chronic inflammation—while advancing the broader vision of responsive, regenerative, and precision medicine.

Several rapidly developing modalities are expanding the conceptual and technological landscape of cell-mediated delivery, and brief acknowledgment of these advances further strengthens the novelty and completeness of the CMDS framework. Neutrophil-mediated delivery (“neutrophil hitchhiking”) has emerged as a powerful strategy in which nanoparticles leverage neutrophils’ natural recruitment to inflammatory and tumor microenvironments to achieve targeted transport across vascular and stromal barriers [101,102]. Cell-backpack systems, involving polymeric or hydrogel micro-patches affixed to leukocytes without internalization, offer long-lived and programmable delivery vehicles that preserve cellular motility and enable sustained release or immunomodulation in vivo [103,104]. On the peptide–nanocarrier side, advanced amphiphilic CPP-based nanocage systems, such as the PepFect and NickFect families, have demonstrated high efficiency in delivering DNA, siRNA, mRNA, and Cas9 RNPs through self-assembled, endosomolytic nanostructures [105]. Finally, complementary carrier-free protein delivery technologies such as iTOP (induced transduction by osmocytosis and propanebetaine) further expand intracellular delivery capabilities, enabling efficient cytosolic entry of recombinant proteins and genome-editing complexes without conventional vectors [106]. Together, these emerging approaches illustrate the accelerating diversification of CMDS technologies and highlight future directions where biological carriers, engineered peptides, and synthetic nanostructures converge into increasingly adaptive and clinically scalable therapeutic systems.

## Figures and Tables

**Table 1:** Summary of preclinical peptide-based delivery systems evaluated for efficacy, toxicity, scalability, and translational readiness.

References	Efficacy	Safety	Scalability	Clinical Translation Stage
[46]	Very high transfection efficiency; NK1R-targeted; crosses BBB in vitro and in zebrafish; P-02 is optimal.	Low cytotoxicity; no in vivo toxicity in zebrafish; SP competition does not introduce toxicity.	SPPS-based modular peptides, high purity, stable self-assembled nanoparticles–good theoretical scalability, but not evaluated.	Early preclinical (cell lines + zebrafish). No mammalian in vivo efficacy or toxicology yet.
[47]	Strong, selective transfection in HER2/3-overexpressing MDA-MB-453 breast cancer cells. No measurable transfection in HER2-low MDA-MB-231 cells, confirming receptor-dependent uptake.	MTT assays show no detectable cytotoxicity in either MDA-MB-453 or MDA-MB-231 at the tested charge ratios.	No discussion of industrial scale-up, formulation stability, or manufacturability beyond lab-scale bacterial expression.	Entirely in vitro work (breast cancer cell lines). No in vivo studies, no pharmacokinetics, no toxicology, no animal tumor models.
[48]	RALA efficiently condenses p53-encoding plasmid DNA at N/P ≥ 2, forming stable nano-sized complexes. Strong intracellular delivery and nuclear accumulation in HeLa cells at N/P 5–10.	Non-toxic to normal fibroblasts across N/P 1–10; viability > 90% at 24–48 h. In HeLa cells, reduced viability results from p53-induced apoptosis, not formulation toxicity.	RALA peptide synthesized via standard solid-phase peptide synthesis (SPPS). Methods rely on routine laboratory procedures (DLS, centrifugation), suggesting straightforward scalability, although not tested at production scale.	Fully in vitro study (HeLa cancer cells + fibroblasts). No in vivo animal studies, no pharmacokinetics, no toxicology.
[49]	CPPs improve antigen uptake, processing, and presentation by APCs. Enhance both humoral and cellular immunity (Th1 bias, CTL activation). Improve mRNA and DNA vaccine delivery, increase stability and expression.	CPPs generally show low toxicity and non-immunogenicity in vitro.	CPPs are easy to synthesize, cost-effective, and can be produced in high quantities.	No CPP-based vaccines are yet FDA-approved.
[50]	LAH4 strongly enhances intracellular delivery of proteins and CpG in dendritic cells. Results in potent protective and therapeutic antitumor effects in B16/OVA and B16 melanoma models—significantly prolonged survival and reduced tumor growth.	No in vivo toxicity reported in mice; all effects attributed to immune responses rather than toxicity.	LAH4 is a synthetic amphipathic peptide—readily manufacturable using solid-phase peptide synthesis. Delivery relies on spontaneous complexation; methods are compatible with scalable vaccine production, though no industrial-scale testing is described.	In vitro dendritic cell uptake and cross-presentation assays; multiple in vivo mouse vaccination and tumor challenge models (OVA/B16 and TRP-2/B16). No pharmacokinetic, biodistribution, toxicology, or GMP studies.
[51]	Efficient intracellular delivery of E7 into HEK-293T cells at 1–3 h post-transfection; none detected with E7 alone. Tumor model: E7/Pep-1 (1:20) provides 80% tumor-free survival, comparable to E7 + Freund’s adjuvant.	MTT assays show no significant cytotoxicity of Pep-1 or E7/Pep-1 nanoparticles at 24–48 h in HEK-293T cells; complexation reduces Pep-1 cytotoxicity.	Pep-1 is a synthetic peptide produced by standard peptide synthesis (SPPS).	Preclinical, early stage. Study includes in vitro cell delivery and in vivo mouse tumor-challenge model. No pharmacokinetics, toxicology, or large-animal studies; no clinical trials reported.
[52]	Effective for tumor targeting, imaging, vaccination, intracellular delivery, and protein delivery (e.g., rhBMP-2 maintained >95% bioactivity when encapsulated.	Peptidomimetic conjugation can increase interaction with immune system–may increase phagocytosis if unmodified.	Most systems rely on synthetic polymer self-assembly, SPPS peptide synthesis, and standard conjugation chemistries (e.g., NHS, maleimide.	No clinical trials involving these peptidomimetic self-assembled systems are reported.

**Table 2:** Summary of Mechanisms and Translational Readiness of Endosomal Escape Strategies for Cell-Based Delivery Systems.

References	Efficacy	Safety	Scalability	Clinical Translation Stage
[53]	In vivo: MN@RMNCs achieve complete tumor ablation, prevent recurrence, and significantly improve survival. cRGD-engineered MN@RMNCs show 1.7 × higher tumor accumulation than unmodified MN@MNCs and 2.3 × higher than free NPs.	In vivo toxicity evaluation shows: no mortality, no weight loss, and normal liver/kidney biochemistry (ALT, AST, ALP, ALB, CRE, UA, URE) across groups.	Whole system relies on established techniques: SPPS peptides, DSPE-PEG carriers, immune cell culture	In vitro: uptake, ROS generation, PDT/PTT cytotoxicity. In vivo: mouse 4T1 breast tumor model; imaging, biodistribution, toxicity, treatment efficacy. No pharmacokinetics, GLP toxicology, or large-animal studies. No clinical trials reported.
[54]	In vivo melanoma model: tumor proliferation reduced to 1.13% of saline control; highest survival with complete tumor suppression.	No histological damage or acute toxicity reported in treated animals.	All steps are widely used in nanomedicine manufacturing—high theoretical scalability, though not validated at GMP/industrial scale.	Data include in vitro melanoma cytotoxicity, NK-cell activation assays, and in vivo xenografted A375 mouse tumor model. No pharmacokinetic, biodistribution, GLP toxicology, or large-animal studies beyond mice. No clinical trials reported.
[55]	Tat and C105Y CPPs effectively deliver cargoes (TAMRA dye; avidin protein) into mast-cell line RBL-2H3 without triggering exocytosis. Cargoes can be released on demand via mastoparan or physiological IgE-mediated activation–demonstrates controlled secretion capability.	Human mast cells may differ substantially in sensitivity to CPP-mediated activation; safety is not yet established.	Major scalability bottleneck is not the CPPs, but the difficulty/cost of isolating, maintaining, and expanding primary human mast cells.	Conceptual early-stage research; no animal studies, no human data. Current data generated only in rat basophilic leukemia (RBL-2H3) mast-cell line.
[56]	Gene complexes (GCs) show ~100% cellular uptake in HUVECs. MMP-triggered release achieves ~40% cumulative release in 24 h, none without enzyme.	NPs and GCs show >80% cell viability at 20 μg/mL. In vivo: no reported adverse effects; no thrombus formation on fully endothelialized graft; normal vessel morphology at 28 days.	Moderate scalability, but manufacturing complexity is higher than single-step peptide systems.	Promising for vascular graft endothelialization but not yet at translational/clinical readiness.

**Table 3:** Summary of Mechanistic Comparison of Endocytic and Non-Endocytic Cellular Uptake Pathways of Peptide CMDS hybrid systems.

References.	Efficacy	Safety	Scalability	Clinical Translation Stage
[57]	Several nanoparticle systems (e.g., transferrin-targeted liposomes, angiopep-2 conjugates) show significant survival improvement in animal glioma models.	Some carriers (liposomes, PLGA, SLNs, dendrimers) are generally recognized as biocompatible, but long-term CNS safety is not well established.	Formulations based on liposomes, PLGA nanoparticles, micelles, and SLNs are considered highly scalable because they use established industrial manufacturing (emulsification, solvent evaporation, microfluidics).	Most technologies remain in early preclinical development (in vitro, rodent models) with very few in clinical trials.
[58]	Nanomaterials (liposomes, polymeric NPs, inorganic NPs, micelles) show enhanced BBB penetration, enabled by receptors (TfR, LfR, LRP1), CPPs, shuttle peptides, intranasal delivery, or temporary BBB-opening	Nanomaterials (liposomes, polymeric NPs, inorganic NPs, micelles) show enhanced BBB penetration, enabled by receptors (TfR, LfR, LRP1), CPPs, shuttle peptides, intranasal delivery, or temporary BBB-opening. Several nanomaterials demonstrate good biocompatibility and are biodegradable.	Many platforms rely on established scalable methods: polymeric NP self-assembly, liposome formation, PEGylation, peptide synthesis.	Most strategies remain preclinical, tested in vitro (BBB cell models) and in vivo (mouse, rat, zebrafish) models
[59]	Multiple BBB-crossing strategies (nanotechnology, hyperthermia, receptor-mediated transport, CPPs, cell-mediated delivery) with strong preclinical evidence of enhanced drug penetration into brain parenchyma.	Nanoparticles may induce inflammatory responses, neutrophil influx, and mortality at high doses	Cell-based delivery has lowest scalability, requiring expansion and engineering of millions of viable stem cells.	No strategy has yet demonstrated major clinical benefit, and most remain preclinical to early clinical stage.
[60]	CPP–siRNA duplexes demonstrated successful delivery to primary neurons in vitro and CNS tissue in vivo	Need to ensure complex stability and control unintended interactions	CPPs are short peptides (10–30 aa) and easily synthesized using standard peptide synthesis, suggesting good manufacturing scalability	Early preclinical, with limited clinical progress limited to invasive delivery strategies.
[61]	PEG12-KL4 efficiently mediates siRNA knock-down of EGFR and PD-L1 in NSCLC lines (NCI-H292, HCC827, NCI-H1975)–reducing EGFR to < 20–40% and PD-L1 to ~50% depending on the cell line	PEGylated KL4 is previously shown to have good pulmonary tolerance in mice. Confocal images and cell viability experiments do not indicate cytotoxicity from the peptide itself (only siRNA-mediated effects).	High theoretical scalability, supported by existing clinical-grade KL4 surfactant experience.	Early preclinical feasibility study, next steps = therapeutic evaluation in NSCLC mouse models.
[62]	Strong preclinical efficacy consistent across rodent AD models: improved cognition, reduced Aβ aggregation, reduced neuroinflammation, inhibition of tau hyperphosphorylation. No human efficacy demonstrated.	Preclinical data suggests acceptable safety for many polymeric systems, but long-term CNS safety remains a major unresolved challenge.	Generally scalable for polymeric/lipid NPs, but metal/bioconjugate NPs may face manufacturing, reproducibility, and regulatory barriers.	All systems remain preclinical. Some patented formulations exist, but no clinical translation or human trials yet.
[63]	Efficacy varies by platform. ACT is described as the most potent; peptide vaccines lag behind.	Safety is acceptable for most platforms, but intensive regimens (ACT) carry known toxicities.	Peptide vaccines are most scalable; ACT/APC vaccines least scalable.	Strongest translation: APC vaccine (approved). ACT is in late-stage trials but not approved. Peptide vaccines are emerging.
[64]	ESCRT-engineered EVs successfully deliver antigenic peptides (OVA) into dendritic cells.	Effective induction of CTL activation shown in vitro. This study does not include in vivo safety data.	Method is currently laboratory-scale and requires co-transfection steps.	In vitro only: no animal vaccination or tumor model. No clinical trial indications.

**Table 4:** Overview of Peptide-Enabled and Cell-based Cancer Therapies in Ongoing Clinical Trials.

Modality/Product	Indication	Trial ID	Phase	Notes
p28 (NSC745104), tumor-targeting CPP	Advanced solid tumors/pediatric CNS tumors	NCT00914914	I	Azurin-derived CPP; preferentially enters cancer cells, stabilizes p53; good tolerability in adults and children. (ClinicalTrials.gov)
IFN-γ–Dex (DC-derived exosomes with tumor Ags)	Maintenance immunotheray in NSCLC	NCT01159288	II	Autologous DC-derived exosomes loaded with MHC I/II tumor antigens after induction chemotherapy. (ClinicalTrials.gov)
DCVax-L (autologous tumor lysate–pulsed DCs)	Newly diagnosed glioblastoma	NCT00045968	III	Autologous DC vaccine loaded with tumor lysate; represents an advanced DC-based, peptide-loaded cell product. (PMC)
DC vaccine for MSI-positive CRC /Lynch syn	Microsatellite instability–positive colorectal cancer	NCT01885702	I/II	Autologous peptide/tumor antigen–loaded DC vaccine in hereditary cancer predisposition. (ClinicalTrials.gov)
Certepetide (LSTA1) + gemcitabine/nab-paclitaxel	Pancreatic ductal adenocarcinoma	NCT05042128	IIb/III	Tumor-penetrating peptide that enhances intratumoral delivery of co-administered chemotherapeutics. (ClinicalTrials.gov)

## Data Availability

No new data were created or analyzed in this study. Data sharing is not applicable to this article.

## Abbreviations

The following abbreviations are used in this manuscript:

ATMPs	Advanced Therapy Medicinal Products
AI	Artificial Intelligence
BBB	Blood-Brain-Barrier
CF-SPPS	Continuous-Flow Solid-Phase Peptide Synthesis
CPPs	Cell-penetrating peptides
CQAs	critical quality attributes
DCs	Dendritic Cells
ECs	Endothelial Cells
ESCRT	Endosomal Sorting Complexes Required for Transport
EMA	European Medicines Agency
FDA	Food and Drug Administration
GMP	Good Manufacturing Practices
HTS	High-Throughput Screening
ICH	International Council for Harmonisation
IMPs	Immune-Modulating Peptides
MD	Molecular Dynamics
ML	Machine Learning
MMP	Matrix Metallo Proteinase
NIR	Near-Infra Red
NK1R	Neurokinin-1 Receptor
RGD	Arginine–Glycine–Aspartic acid motif
REDV	Arg–Glu–Asp–Val motif
QbD	Quality-by-Design
PAAs	Polyacrylates
RALA	Ras-Related Protein
TAT	Trans-Activator of Transcription
